# Cytokine Hyperresponsiveness in Children With ETV6::RUNX1-positive Acute Lymphoblastic Leukemia After Challenge With Common Pathogens

**DOI:** 10.1097/HS9.0000000000000835

**Published:** 2023-01-31

**Authors:** Nadine Rüchel, Marina Oldenburg, Stefan Janssen, Aleksandra A. Pandyra, Wei Liu, Eleni Vasileiou, Daniel Hein, Vera Helena Jepsen, Ute Fischer, Daniel Picard, Gesine Kögler, Julia Hauer, Franziska Auer, Angelina Beer, Ortwin Adams, Colin MacKenzie, Martin Jaeger, Mihai G. Netea, Arndt Borkhardt, Katharina L. Gössling

**Affiliations:** 1 Department of Pediatric Oncology, Hematology and Clinical Immunology, Medical Faculty, Heinrich-Heine University Düsseldorf, Germany; 2 Algorithmic Bioinformatics, Department of Biology and Chemistry, Justus Liebig University Gießen, Germany; 3 Molecular Medicine II, Medical Faculty, Heinrich-Heine University Düsseldorf, Germany; 4 DKTK, Partner Site Essen/Düsseldorf, Germany; 5 Department of Pediatric Neuro-Oncogenomics, DKFZ, Heidelberg, Germany; 6 Institute of Neuropathology, Medical Faculty, Heinrich-Heine University Düsseldorf, Germany; 7 Institute for Transplantation Diagnostics and Cell Therapeutics, Medical Faculty, Heinrich-Heine University Düsseldorf, Germany; 8 Pediatric Hematology and Oncology, Department of Pediatrics, University Hospital Dresden “Carl Gustav Carus,” TU Dresden, Germany; 9 Department of Pediatrics, Children’s Cancer Research Center, Kinderklinik München Schwabing, School of Medicine, Technical University of Munich, Germany; 10 Institute for Virology, Medical Faculty, University Hospital and Heinrich-Heine University Düsseldorf, Germany; 11 Institute of Medical Microbiology and Hospital Hygiene, University Hospital, Heinrich-Heine University Düsseldorf, Germany; 12 Department of Internal Medicine and Radboud Center for Infectious Diseases, Radboud University Medical Center, Nijmegen, the Netherlands; 13 Department of Immunology and Metabolism, Life and Medical Sciences Institute, University of Bonn, Germany

In 1922, Poynton et al formulated the concept that leukemia develops as an abnormal response to infection. Epidemiological and experimental studies of B-cell precursor acute lymphoblastic leukemia (BCP-ALL) support this hypothesis, namely the space-time clusters of childhood BCP-ALL^1,2^ and leukemia-susceptible animal models exposed to infectious agents.^2^ The infectious challenge is considered a secondary event incurred on a pre-existing preleukemic clone, favoring its subsequent outgrowth. Preleukemic clones can be initiated either by germline mutations, cells with acquired fusion genes (eg, ETV6::RUNX1) or chromosomal aneuploidy. We and others have shown that the percentage of healthy newborns who bear such preleukemic clones at birth remain leukemia-free throughout their life is up to 5%.^3^

Genome-wide association studies in BCP-ALL have identified frequent but low-penetrant germline variations.^4^ When the 5 most frequent risk alleles are combined in a polygenic score, the overall risk for developing BCP-ALL is elevated approximately fivefold, but still remains low, since the disease affects 1 of 2000 children below the age of 15. Some of those risk alleles show no obvious connection to immunologically relevant genes. However, immune responses are extremely complex and influenced by underlying genetic and environmental factors.^5^ The aim of this study was to understand whether children with the most common subtypes of BCP-ALL (high-hyperdiploidy and ETV6::RUNX1) present with an aberrant immune response against common pathogens to which children are normally exposed during early childhood. Furthermore, the comparison with the immune profile of cord blood carrying the ETV6::RUNX1-positive (ETV6::RUNX1+) clone should help to decipher the specificity for children, who develop ALL or stay healthy despite the presence of the preleukemic clone at birth.

Thus, we analyzed the presumed “abnormal immune response” at a functional level by comparing the stimulated peripheral blood mononuclear cells (PBMCs) of children with BCP-ALL and their parents with that of healthy counterparts (Suppl. Materials and Methods). Bacterial, viral, and fungal components and activators of the innate pattern recognition receptor signaling pathways were used to cover a broad variety of stimuli. In our approach, PBMCs were stimulated (Figure 1) either short term, to measure the monocyte-derived cytokines, or long term, to measure the T helper-derived cytokines.^6^

**Figure 1.**
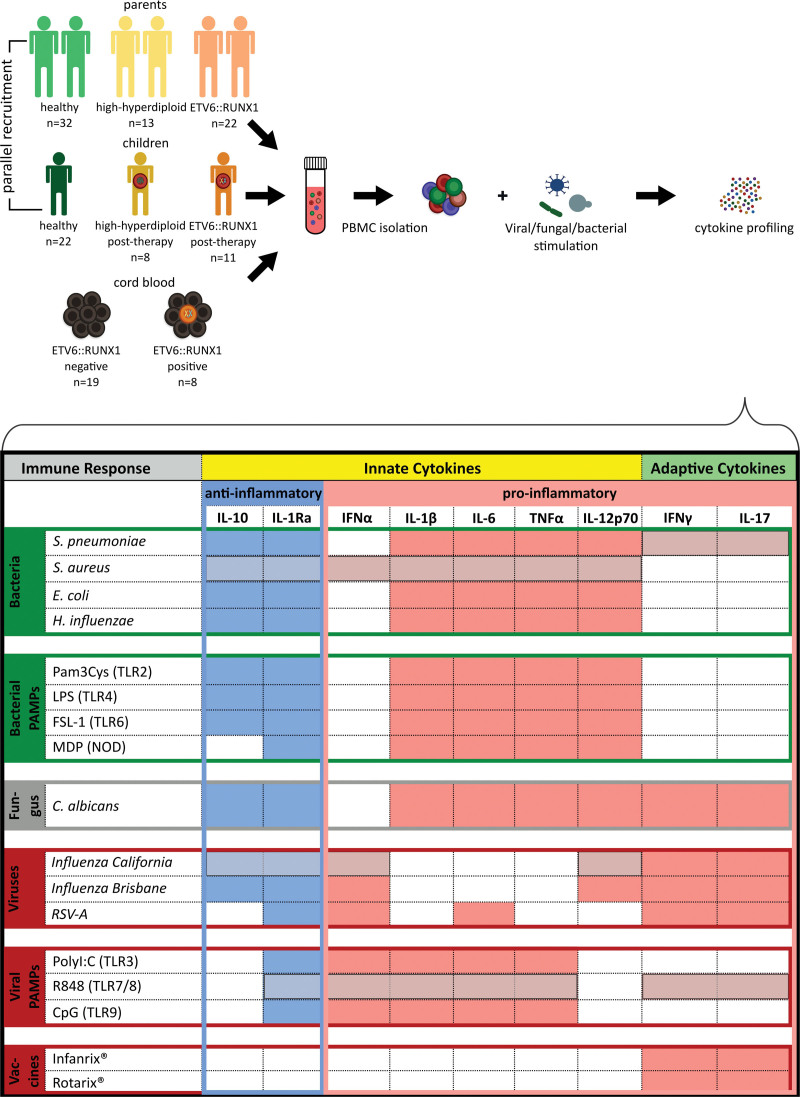
**Overview of the study design.** BCP-ALL families were recruited in parallel with healthy families, and ETV6::RUNX1-positive cord blood was stimulated in parallel to ETV6::RUNX1-negative cord blood. After isolation of PBMCs, cells were stimulated with various infectious stimuli to perform the cytokine profiling via measurement of cytokines in the cell culture supernatant. The stimulation panel consisting of whole bacteria, viruses, PAMPs and vaccines was classified into the following different immune response characteristics: anti- (blue column) vs proinflammatory (light red column) cytokines, antibacterial (green rows), antiviral (dark red rows) and antifungal (gray row) immune responses. Cord blood cells were stimulated with a reduced stimulation panel due to limited cell numbers (light gray). BCP-ALL = B-cell precursor acute lymphoblastic leukemia; PAMPs = pathogen-associated molecular patterns; PBMCs = peripheral blood mononuclear cells.

Cytokine responses underlie a large genetic-, environmental-, life-style-, and stimulus-specific variability.^5^ Thus, we carefully recruited an age-, sex-, and BMI-matched control cohort in parallel to the patients’ cohort to average out the effects of season, age, sex and BMI. In total, 37 families consisting of 101 individuals were recruited within 2 years, including 11 children with cured BCP-ALL and ETV6::RUNX1 fusion with their parents, 8 children with cured high-hyperdiploid BCP-ALL with their parents and 18 healthy children with their parents (Suppl. Tables S1 and S3). All patients had been enrolled in the ALL-BFM 2017 or Co-ALL 08/09 therapy protocols and were treated in the German Pediatric University Center of either Dü (sseldorf or Dresden. (Suppl. Table S2). All children were in complete remission, without any sequelae and at least two years past cessation of maintenance therapy.^7^ Immunophenotyping excluded significant differences in the cellular composition of the peripheral blood (Suppl. Figure S1A–C). To study the functional immune response, we used an immune profiling protocol established for the Human Functional Genomic Project as used in other functional genomic studies.^6^ Isolated PBMCs were stimulated followed by subsequent measurement of cytokines in the cell culture supernatant (100 biologically relevant stimulus-specific cytokines).^8^ We clustered either cytokines or stimuli into different features (antibacterial, antifungal, and antiviral immune response) and compared patients with their controls (Figure 1). Moreover, 8 frozen cord blood samples with proven ETV6::RUNX1 fusion detected by the genomic inverse PCR for exploration of genomic breakpoints (GIPFEL) technique were included, as well as 19 cord blood samples without ETV6::RUNX1+ cells. None of these ETV6::RUNX1+ samples had developed leukemia.

While baseline cytokine response of children with successfully treated BCP-ALL did not differ from that of healthy children (Figure 2A), they had a markedly elevated overall cytokine response (q < 0.01) to infectious stimuli (Figure 2B and C). This elevated response was not observed in children cured from a high-hyperdiploid karyotype BCP-ALL (Figure 2D) but specifically in children cured from ETV6::RUNX1+ BCP-ALL (Figure 2E). Segregating the different categories of viral, fungal and bacterial stimuli, we found viral and bacterial triggers were responsible for this observation (q < 0.05) (Figure 2F and G), while fungal triggers did not contribute to increased cytokine production (Figure 2H). Further separation of the immune responses into cytokine-stimulus-pairs resulted in 3 potential candidates: R848-induced IL-17 and IFNα responses and *Influenza California*-induced IFNα production, whose significance (*P* < 0.05) was lost due to correction for multiple testing (q > 0.05) (Figure 2I). The ETV6::RUNX1 fusion is frequently acquired during fetal hematopoiesis. Thus, we asked whether this cytokine response pattern could also be seen in healthy carriers of the ETV6::RUNX1 fusion at birth (Figure 2J). We stimulated cord blood containing ETV6::RUNX1+ cells (n = 8) with the most promising stimuli candidates and compared it to cord blood containing no ETV6::RUNX1+ cells (n = 19) (Figure 2J). There was no difference in cytokine production between these groups, suggesting that the fraction of ETV6::RUNX1+ cells (ranging from 7.1 × 10^−5^ to 4.6 × 10^−3^) is not responsible for the functional hyperresponsiveness (Figure 2I and J).

**Figure 2.**
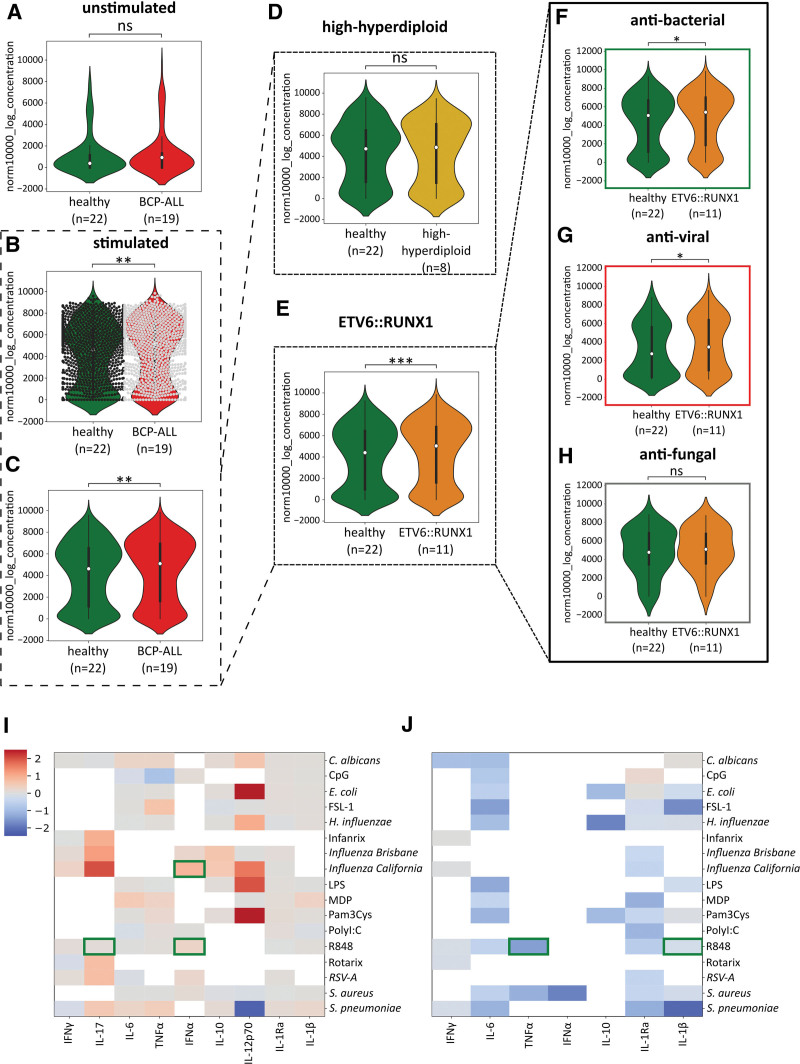
**The functional immune response is specifically elevated in ETV6::RUNX1-positive BCP-ALL patients, but not in ETV6::RUNX1-positive cord blood.** PBMCs were stimulated with different classes of antigens (bacteria, fungi, viruses). Cytokines were measured in the cell culture supernatant by ELISA. Comparison of the (A) unstimulated and (B and C) stimulated general immune response of all BCP-ALL patients and control children, (D) high-hyperdiploid patients and control children and (E) ETV6::RUNX1-positive BCP-ALL patients and control children, further distributed into (F) antibacterial, (G) antiviral, and (H) antifungal cytokine response. (B) Individual data points were plotted to show the number of actual concentrations in (C) (1174 for healthy and 1174 for BCP-ALL). A pattern of the immune response of (I) ETV6::RUNX1-positive BCP-ALL patients (n = 11) vs control children (n = 22) and (J) ETV6::RUNX1-positive (n = 8) and negative cord blood cells (n = 19) was distributed into the different cytokine-stimulus pairs using a heatmap showing the log FC of cytokine-stimuli pairs. Green boxes indicate significant *P* values (<0.05) before correction. For the white fields no cytokines were detectable. Cytokine concentrations were normalized and logtransformed. (A–J) Statistical analysis was performed using Mann–Whitney–Wilcoxon test two-sided with Benjamini-Hochberg correction for multiple testing. A q-value of <0.05 after correction was considered statistically significant (*q < 0.05, **q < 0.01, and ***q < 0.001). BCP-ALL = B-cell precursor acute lymphoblastic leukemia; FC = fold change; PAMPs = pathogen-associated molecular patterns; PBMCs = peripheral blood mononuclear cells.

Cytokine responses are both genetically determined and substantially influenced by environmental factors, such as gender, season or environment.^5,8^ Next, we asked about the influence of cohabitation, and integrated the parents into the experimental setup. We analyzed the cytokine response of their PBMCs and compared the cytokine response phenotype within biological families to randomly shuffled families (Suppl. Figure S2A). Biological families are much more similar in terms of their cytokine response pattern than artificially generated randomly shuffled families (Suppl. Figure S2A), attributing this to different life-style factors. Children showed a significant higher similarity to the mothers’ profiles compared to the fathers’ profile (Suppl. Figure S2B) in all families independent of the disease (Suppl. Figure S2C).

Comparing the parents of the ETV6::RUNX1 patients with their healthy counterparts revealed no differences in the unstimulated setting (Suppl. Figure S3A). Furthermore, we could not detect an overall elevated cytokine response, when all stimuli were combined in the analysis (Suppl. Figure S3B), in parents from children with high-hyperdiploid BCP-ALL (Suppl. Figure 3C), or in parents of ETV6::RUNX1+ children (Suppl. Figure S3D). Segregation into antiviral, antibacterial, and antifungal immune responses also revealed no significant differences (Suppl. Figure S3E–G). On the one hand, biological TRIOs shared the same cytokine profile, while on the other hand the parents with BCP-ALL children did not differ from their children. This means that the cytokine profile seems to be quite specific to those children, who had developed an ETV6::RUNX1-positive leukemia and seems to be influenced by factors other than environmental factors.

Functional experiments revealed a partial response to infectious stimuli in terms of higher cell death and decreased proliferation of the B-cell precursor leukemia NALM-6 cell line stably expressing ETV6::RUNX1 (Suppl. Figure S6). Short term *ex vivo* stimulation of bone marrow cells from Sca1-ETV6::RUNX1 mice did not result in a shift in the immunophenotype or a different cytokine production profile (Suppl. Figures S4 and S5). These results are in line with the very low probability of these mice developing leukemia during their life-span.^2^

Like other diseases, the development of BCP-ALL in children involves multiple genetic, immunological and environmental factors and studying their complex interplay remains a challenge. Measuring individual, stimulus-induced cytokine responses was shown to be very informative with regards to the impact of genetic and nongenetic host factors on the variability of baseline immune responses.^5,8^ The impact of an altered cytokine milieu on the outgrowth of preleukemic cells, and in particular ETV6::RUNX1-expressing cells, has been demonstrated in in vitro models using hematopoietic progenitor cells.^9–11^ In addition, aberrant cytokine levels have been detected at birth in dried blood spots from newborns who later developed BCP-ALL.^12,13^ Both genetic subtypes tested in our study are initiated in utero during fetal hematopoiesis and represent the 2 most frequent genetic forms in children.^14^ However, they do not necessarily share common biological mechanisms and even their cell of origin may be different. Upon stimulation with *Influenza* antigens, we uncovered a trend toward higher IFNα-production, a key cytokine whose chronic activation impairs hematopoietic stem cells (HSCs) in mice, while acute exposure can lead to activation and proliferation of dormant HSCs and early progenitor cells.^15^ IFNα also activates JAK-STAT-signaling, a crucial pathway in the development of BCP-ALL whose timely inhibition may even prevent the development of BCP-ALL in mouse models with genetic leukemia predisposition.^2^

The main limitation of the study is the small number of patients that can only partially be compensated by the broad cytokine panel and the detailed functional analysis. Nevertheless, the study had been performed with an unbiased screening approach to unravel candidate cytokines and stimuli for further investigation and functional validation of their role in leukemogenesis.

In summary, we show that PBMCs from children with ETV6::RUNX1 BCP-ALL globally produce higher levels of cytokines when triggered with viral and bacterial, but not fungal stimuli. Future long-term, prospective studies may help to clarify whether this functional hyperresponsiveness allows the development of strategies for predicting which children are at high risk for developing ETV6::RUNX1 + BCP-ALL.

## ACKNOWLEDGEMENTS

We appreciate the participation and blood donation from all of our patients, their parents and healthy volunteers. The authors thank Almuth Düppers, Lutz Körschgen, Stefanie Liedtke, Heidi Seliger, Birgit Ackermann and Bianca Killing for experimental help, as well as Stewart Boden for the careful proofreading.

## AUTHOR CONTRIBUTIONS

Contribution: MGN, KLG, JH, FA, AB, and AB conceived and designed the project; AB, KLG, and AB recruited the patients and volunteers; NR, MO, SJ, KLG, MJ, MGN, and AB developed the methodology; NR, MO, SJ, AAP, EV, KLG, and AB were responsible for analyzing and interpreting the data (statistical analysis, biostatistics, computational analysis); NR, KLG, and AB prepared the manuscript; JH, FA, AAP, WL, EV, DH, VHJ, UF, GK, OA, CM, MJ, MGN, and DP provided administrative, technical, experimental, or material support (reporting or organizing data, constructing databases).

## DISCLOSURES

The authors have no conflicts of interest to disclose.

## SOURCES OF FUNDING

The German Research Foundation (Deutsche Forschungsgemeinschaft, DFG, Grant No. 428917761) supported K.L.G. ERC Stag 85222 PreventALL supported JH. The work was supported by the Katharina-Hardt-Stiftung, the Christiane und Claudia Hempel Stiftung, and Löwenstern e.V. A.B. and U.F. (FKZ: 3618S32275 and 3618S32274) were supported by the German Federal Office for Radiation Protection (BfS). A.B. (DJCLS 07/19) and U.F. were funded by the German Carreras Foundation (DJCLS 18R/2021, DJCLS 21 R/2019). U.F. was funded by the Deutsche Forschungsgemeinschaft (DFG, German Research Foundation—Projektnummer 495318549) and the German Cancer Aid (Priority Program Cancer Prevention—Graduate School). AAP was supported by the Research Commission of the Medical Faculty of the HHU (2021-04), the José Carreras Foundation (DJCLS 07 R/2019), the Ilsedore Luckow Stiftung and the German Federal Office for Radiation Protection (BfS).

## Supplementary Material


