# Supplementary material for: Cytokine Hyperresponsiveness in Children With ETV6::RUNX1-positive Acute Lymphoblastic Leukemia After Challenge With Common Pathogens
Source: Hemasphere. 2023 Jan 31;7(2):e835. doi: 10.1097/HS9.0000000000000835 (PMC9891444; doi:10.1097/HS9.0000000000000835)
Supplement: Supplementary file 1 [file hs9-7-e835-s001.docx]

**Supplemental Section:**

**SDC, Material and methods:**

*Sample collection and control for experimental variables*

We recruited families with a child previously diagnosed with either a high-hyperdiploid karyotype or an ETV6::RUNX1 fusion gene BCP-ALL (Figure 1). Children were diagnosed and treated according to the ALL-BFM multicenter trial in two pediatric oncology university centers, Düsseldorf and Dresden. Karyotyping and routine genetic analysis were done in the respective reference laboratories at the Medical School Hannover. We enrolled an age- and sex-matched healthy control cohort recruited in parallel, mostly from volunteering nurses and physicians with a matching family. To minimize experimental variables, patient and control samples were collected and measured in a highly standardized manner. Stimulation experiments were always performed the same day, by the same investigators (MO, NR). Former BCP-ALL patients had no evidence of disease, were free of late sequelae and fully integrated into their social lives. Maintenance therapy had been completed at least two years before enrollment into the study.

After written informed consent, all experiments were performed in accordance to good clinical practice, the Declaration of Helsinki, and with the approval of the Ethical Committee of the Medical Faculty of Heinrich-Heine University in Düsseldorf or the Ethical Committee of the Technical University Dresden.

*Flow cytometry*

Blood count test was performed on fresh whole blood and extracellular staining was performed using CD4-FITC, CD45RO-PE, HLA-DR-ECD, CD56-PC5.5, TCRgd-PC7, TCRab-APC, CD45RA-APC A750, CD3-PacBlu, CD8-KrO, IgD-FITC, CD14-ECD, CD27-PC5.5, CD25-PC5.5, CD20-PC7, CD40-APC, CD127-APC, CD66b-APC A750, CD19-PacBlu, and CD45-KrO antibodies (BD Biosciences, Brea, USA). Counts were measured with a Navios flowcytometer and data were analyzed using CXP analysis software v2.2 (BD Biosciences, Brea, USA).

*PRR ligands and other stimuli*

Stimuli were used in the following concentrations: Bacterial stimuli: *Streptococcus (S.) pneumoniae* clinical isolate (1x10^7^ CFU/ml), *Staphylococcus (S.) aureus* clinical isolate *(*1x10^6^ CFU/ml*), Escherichia (E.) coli* clinical isolate (1x10^7^ CFU/ml), and *Haemophilus influenzae* *(H. influenzae)* clinical isolate (1 x10^7^ CFU/ml), kindly provided by Prof. Mihai Netea (Radboudumc Nijmegen, The Netherlands) and by Prof. MacKenzie (Department of Microbiology, University Hospital Düsseldorf). Viral stimuli: heat-inactivated *Influenza California* (*Infl. California*, 3.2 x10^5^ K/ml TCID50), heat-inactivated *Influenza Brisbane* (*Infl. Brisbane*, 7.4 x10^3^ K/ml TCID50), and *Respiratory Syncytial Virus* (*RSV)-A* (1x10^6^ K/ml TCID50) were prepared as previously described. The fungal stimulus, *Candida albicans* ATCC MYA-3573 (UC820) yeast (1x10^6^ CFU/ml), was prepared as previously described. Pathogen associated molecular patterns (PAMPs): PolyIC (100 µg/ml; TLR3 ligand), R848 (3 µg/ml; TLR7/8 ligand), ODN2006 (unmethylated CpG dinucleotides; 10 mM; TLR9 ligand), and FSL-1 (1 µg/ml; TLR2/6 ligand) were all from Invivogen, San Diego, USA. Pam3Cys (10 μg/ml; TLR2 ligand, EMC microcollections, Germany), N-acetylmuramyl-ananyl-D-isoglutamine (MDP; 10 μg/ml; NOD2 ligand) and *E. coli* lipopolysaccharide (LPS; 10 ng/ml; TLR4 ligand, *E. coli* serotype O55:B5, Sigma-Aldrich, St. Louis, USA). Vaccines: The commercially available vaccines against Pertussis, Polio, Diphtheria, Haemophilus Influenzae B, Tetanus, Hepatitis B (Infanrix hexa®) and Rotavirus (Rotarix®) were obtained from the pharmacy and used in a 1:400 dilution, which had been tested in a serial dilution for stimulatory activity.

*PBMCs isolation and stimulation experiments*

Whole blood samples were diluted 1:1 with Phosphate Buffered Saline (PBS) and subsequently PBMCs were isolated using Ficoll-Paque TM Plus GE (Sigma-Aldrich, St. Louis, USA) density gradient centrifugation. The PBMCs layer was collected and washed twice in cold PBS. Cells were reconstituted in RPMI+ medium (Life Technologies Europe, Bleiswijk, The Netherlands) with 1% penicillin/streptamycine (Sigma-Aldrich, St. Louis, USA, respectively) and counted with a cell counter (Beckmann Coulter, Woerden, Netherlands). PBMCs were plated in a 96-well plate (Corning, NY, USA) at a final concentration of 1.25x10^6^ /ml and 2.5x10^6^ /ml in an end-volume of 200 μl per well for 24h-stimulation to measure the innate cytokines and for 7 day-stimulation to measure the adaptive cytokines, respectively. Stimulations were performed in the presence of 2% human serum (Sigma-Aldrich, St. Louis, USA) for the 24h and in the presence of 10% human serum for the 7 days. Cells were incubated at 37°C with 5% CO_2_, after 24 hours or 7 days, respectively, supernatants were collected and stored at -80°C.

*Cord blood preparation and stimulation*

Fresh cord blood (n=9; 2 ETV6::RUNX1 positive and 7 ETV6::RUNX1 negative) was obtained from the José Carreras Cord Blood Bank, Düsseldorf. Mononuclear cells were isolated as described above using Ficoll-Paque density gradient centrifugation. Cells were frozen using a cryopreservation medium containing 10% DMSO (Sigma-Aldrich, St. Louis, USA) und 90% FCS (Biowest, Nuaillé, France) and stored in liquid nitrogen. Frozen backup samples were used in parallel (n=18; 6 ETV6::RUNX1 positive and 12 ETV6::RUNX1 negative) and were kindly provided by the Institute for Transplantation Diagnostics and Cell Therapeutics (ITZ), University Hospital Düsseldorf. These cells had been cryopreserved using hydroxyethylstarch (HES) and DMSO. After thawing, lysis of erythrocytes was performed using an isotonic ammonium chloride solution. Samples were processed in parallel for the stimulation assays. GIPFEL screening was performed as described previously. On the day of the experiment, ETV6::RUNX1+ and ETV6::RUNX1-negative cord blood samples were defrosted and processed in parallel for the stimulation assays as described above.

*Cytokine measurements*

IL-1β, IL-1Ra, IL-10, IFNα and IL-12p70 were measured in the cell culture supernatants after 24h stimulation using a custom-made multiplex ELISA kit (Procartaplex, Life Technologies GmbH, Darmstadt, Germany), while IL-6 and TNFα were measured using singleplex ELISA (R&D systems, Minneapolis, USA) according to the manufacturer’s instructions. The adaptive cytokines IL-17 and IFNγ were measured in the cell culture supernatant after 7 days stimulation according to the manufacturer’s instructions (R&D systems, Minneapolis, USA). To compare qualitatively different cytokine concentrations with each other we performed normalization to a range between 0 and 10,000 and log-transformation of the raw concentrations as described previously. Raw data, quality control and complete analysis is available at <https://github.com/jlab/microbiome_goessling_cytokine>. Categorization in the anti-bacterial, anti-fungal and anti-viral immune responses is shown in Figure 1.

*Functional analyses – mouse experiments*

Bones harvested from Sca1-ETV6::RUNX1+ or littermate control Sca1-ETV6::RUNX1- mice (backcrossed to the C57BL/6 background for more than nine generations) were flushed and subjected to erythrocyte lysis buffer (0.5 M NH_4_Cl_2_, 10 mM KHCO_3_, 0.1 mM EDTA (pH 7.2)). 3x10^5^ cells per well of a 96 round bottomed plate were plated in RPMI 1640 medium supplemented with 10% LPS-free FCS, 1% antibiotics and 0.1% 50 mM 2-mercaptoethanol. Prior to challenge, eFluor proliferation dye was added. Cells were challenged with R848 (10 µM), LPS (100 ng/ml), heat-inactivated Influenza California (3.2 x10^5^ K/ml TCID50) or recombinant murine interferon α (250 U/ml) (Thermo Fisher Scientific, Massachusetts, USA) for 48 hours after which cells were analyzed using flow cytometry. Apoptosis, proliferation and IL-7 receptor expression were analyzed in the Pre-BI compartment and Pre-BII compartment.

Furthermore, murine IL-6, IL-10, IFNγ and TNFα were measured in the cell culture supernatant after 24 hour and 48 hour stimulation by ELISA (Thermo Fisher Scientific, Massachusetts, USA) according to the protocol provided by the manufacturer.

*Functional analyses – Cell culture experiments*

B cell precursor leukemia cell line NALM-6 cells stably expressing ETV6::RUNX1 or a vector control were stimulated for 24 hours with LPS (50 ng/ml and 100 ng/ml) and PolyI:C (20 µg/ml) at a density of 1x10^6^ /ml, after which they were surface stained with anti-CD80, CD40, CD86, CD127 (IL-7R), HLA-ABC, HLA-DR, CD268 (BAFF-R), CD274 (PD-L1) antibodies (all eBioscience, Thermo Fisher Scientific, Massachusetts, USA).

This cell model was also used for measurements of dead cells and proliferation after stimulation for 24 hours and 72 hours with recombinant human IFNα (100 ng/ml and 400 ng/ml) (Invivogen, San Diego, USA), *Influenza California* (3.2 x10^5^ K/ml TCID50), R848 (3 µg/ml) and *S. aureus* (1x10^6^ CFU/ml). Cells were stained with CFSE (Thermo Fisher Scientific, Massachusetts, USA) according to the protocol provided by the manufacturer before incubation time and seeded at a density of 1.25x10^6^ /ml. After the respective incubation times, cells were stained with DAPI (Sigma-Aldrich, St. Louis, USA).

Measurements were performed using flow cytometry (CytoFlex, Beckman Coulter, Brea, USA) and analyzed using FlowJo v10.8.1 software (BD Biosciences, Brea, USA).

*Statistical analyses*

IFNγ, IL-17, IL-6 and TNFα have been measured using singleplex ELISA. Concentrations were inferred via linear regression (scikit-learn 0.21.3) from four replicated dilution series with seven data points each. All other cytokines have been measured via multiplex ELISA. Their concentrations where directly obtained from the Excel readout file, namely the sheets “Obs Conc”. Values below detection level have been replaced with lowest dilution series concentrations. To control for sample contamination, concentrations two times above the z-score of unstimulated concentrations have been dropped.

Comparisons of concentrations have only been done between samples of the sample measurement day. Throughout this study, we used two-sided Mann-Whitney U tests (scipy 1.3.1) with Benjamini-Hochberg correction for multiple testing (statsmodels 0.12.2). Corrected q-values are considered statistically significant and flagged as * = q < 0.05, ** = q < 0.01 and *** = q < 0.001 and “ns”, otherwise. Note that we indicate “significance” in the heatmaps of Figure 2 based on uncorrected p-values to show trends since no q-value reached significance niveau.

For the mouse experiments, comparisons between more than two groups were performed using a One-way ANOVA with a Dunnett post hoc test, while for analyses between two groups an unpaired t test was used. For the functional analyses with the NALM-6 cell model, one sample t tests were used to compare unstimulated and stimulated conditions and an unpaired t test to compare empty and ETV6::RUNX1 expressing cells.

**SDC, List Tables and Figures**

**SDC, Table. 1 Demographic statistics.** Characterization of the study cohort. Sex, age, height and weight of the ETV6::RUNX1 patients and parents, high-hyperdiploid patients and parents, healthy children and parents. Two healthy control families served as controls twice on different days explaining the slight discrepancy between the numbers in this table and Figure 2.

|  |  | **Sex** | | **Age** | | **Height [cm]** | | **Weight [kg]** | |
| --- | --- | --- | --- | --- | --- | --- | --- | --- | --- |
| **Group** | **total** | **female** | **male** | **[av]** | **[sd]** | **[av]** | **[sd]** | **[av]** | **[sd]** |
| **ETV6::RUNX1 patients** | 11 | 5 | 6 | 11.7 | 4.6 | 149.0 | 23.8 | 50.7 | 29.7 |
| **ETV6::RUNX1 parents** | 22 | 11 | 11 | 43.5 | 7.8 | 173.9 | 8.0 | 83.3 | 19.8 |
| **High-hyperdiploid patients** | 8 | 2 | 6 | 8.4 | 3.0 | 131.5 | 18.8 | 30.2 | 10.1 |
| **High-hyperdiploid parents** | 13 | 8 | 5 | 41.0 | 4.1 | 167.1 | 8.5 | 72.0 | 18.6 |
| **Healthy children** | 18 | 10 | 8 | 9.8 | 6.0 | 139.3 | 29.7 | 38.2 | 21.5 |
| **Healthy parents** | 29 | 14 | 15 | 41.5 | 8.2 | 178.5 | 8.9 | 77.6 | 15.8 |
| **total** | 101 | 50 | 51 | 30.3 | 16.8 | 161.8 | 24.1 | 63.9 | 27.4 |

av = average, sd = standard deviation

**SDC, Table. 2 Medical data of the patients.** Study ID, leukemia genotype, CNS status, treatment protocol, prednisone response on day 8 (PGR = prednisone good response), treatment arm, minimal residual disease (MRD) marker at time points 1 and 2, and complications of all patients are listed. D = Duesseldorf. DD = Dresden. SR = small risk. MR = medium risk. HR = high risk. MRD = minimal residual disease. PGR = prednisone good response.


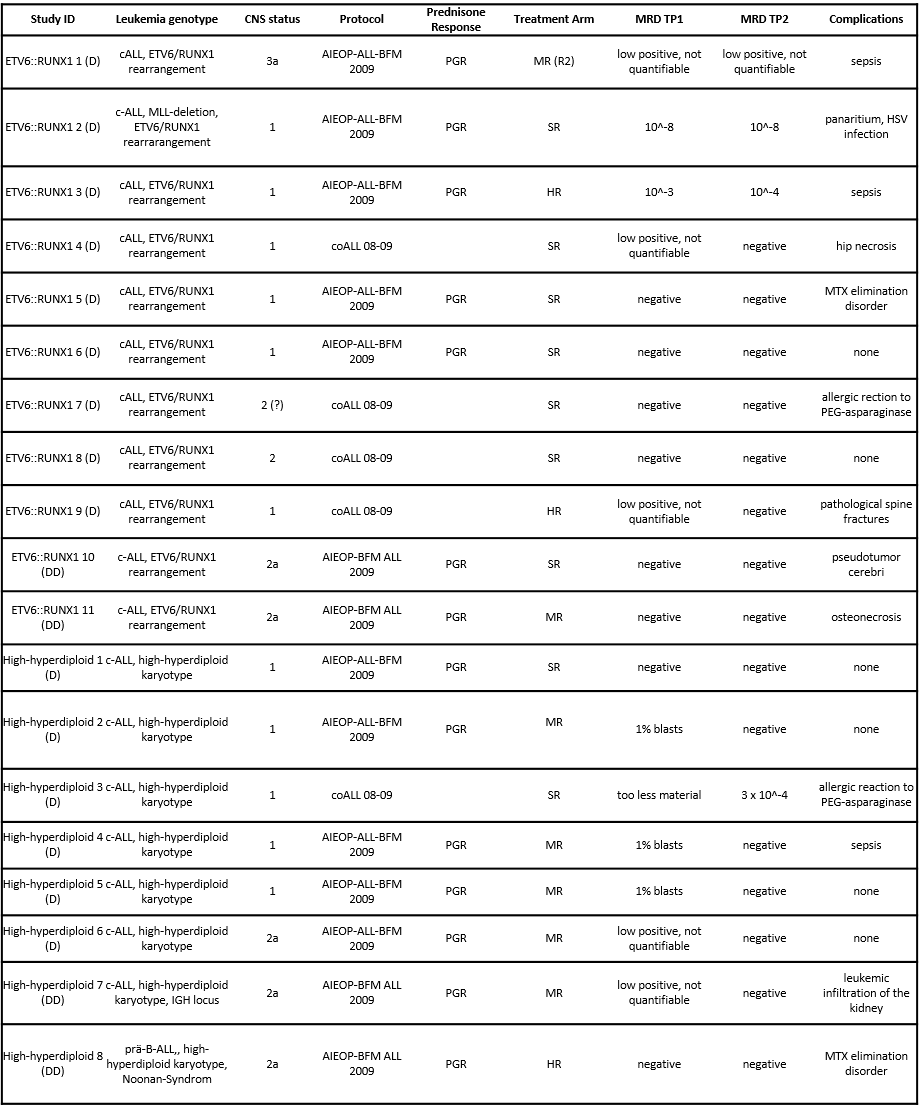


**SDC, Table. 3 Patients’ characteristics at time of diagnosis.** Age, height and weight of ETV6::RUNX1 and high-hyperdiploid patients at time of diagnosis are listed.

|  | **Age** | | **Height [cm]** | | **Weight [kg]** | |
| --- | --- | --- | --- | --- | --- | --- |
| **Group** | **[av]** | **[sd]** | **[av]** | **[sd]** | **[av]** | **[sd]** |
| **ETV6::RUNX1 patients** | 5.5 | 3.4 | 115.3 | 24.4 | 26.5 | 23.7 |
| **High-hyperdiploid patients** | 5.9 | 3.7 | 113.2 | 23.5 | 21.6 | 9.9 |

av = average, sd = standard deviation

**SDC, Figure. 1: BCP-ALL patients do not differ from the healthy controls in their cellular immune phenotype.** (A-C) Unstimulated cells were colored for different immunological surface markers and analyzed using flow cytometry. (A) CD45+ cells. (B) CD19+ cells. (C) CD14+ cells. (A-C) Statistical analysis was performed using Mann-Whitney-Wilcoxon test two-sided with Benjamini-Hochberg correction for multiple testing. A q-value of < 0.05 after correction was considered statistically significant (* = q < 0.05, ** = q < 0.01 and *** = q < 0.001).


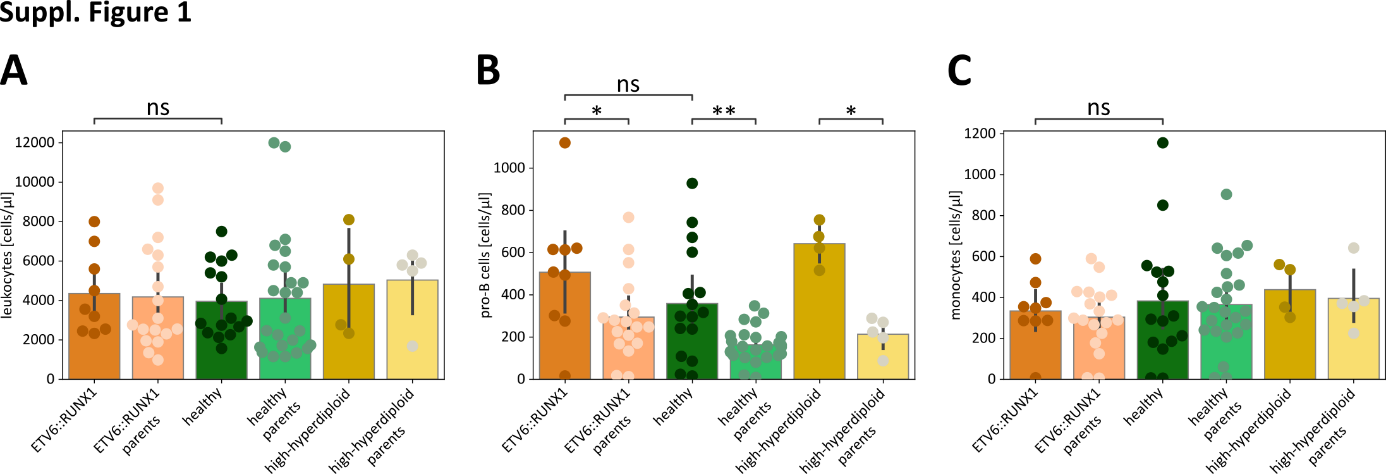


**SDC, Figure. 2: Biological TRIOs significantly differ from shuffled TRIOs, with significant differences in the distance from child to mother compared to child to father, while the interfamilial distances are not dependent whether the child was diseased or not.** Distances of the general immune phenotype between (A) biological TRIOs and shuffled TRIOs, (B) children to the mother or the father, and (C) parents and children within the healthy and BCP-ALL groups were calculated and are depicted as box plots. Cytokine concentrations were normalized and log-transformed. Statistical analysis was performed using two sided Mann-Whitney tests. A p-value of < 0.05 was considered statistically significant (** = p < 0.05, * = p < 0.05).


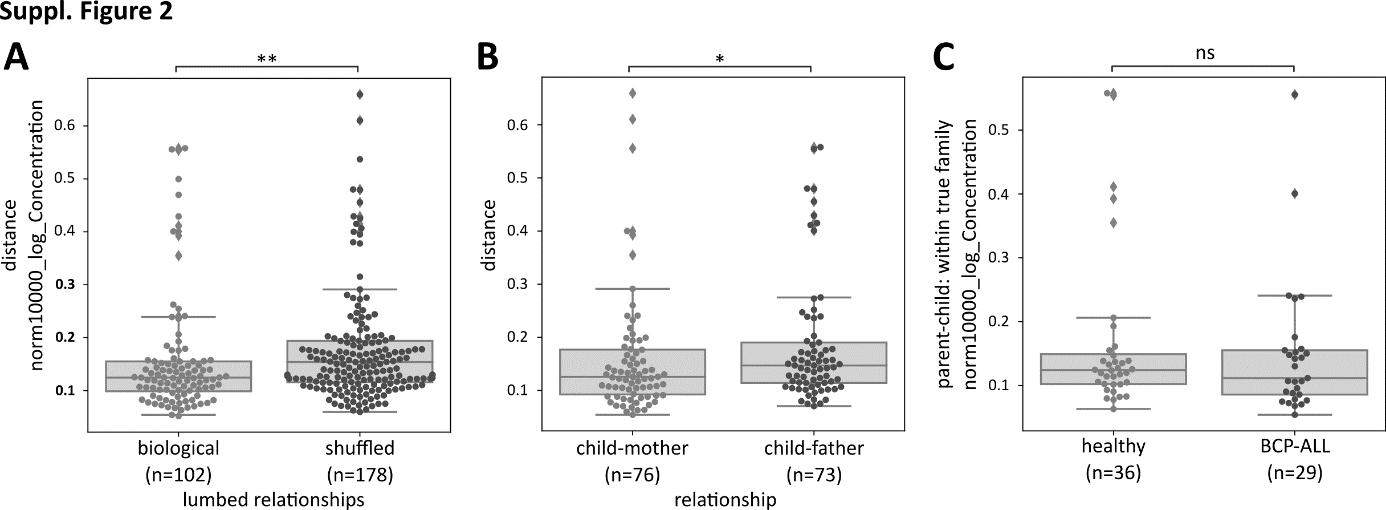


**SDC, Figure. 3: The immune responses of parents of BCP-ALL patients do not qualitatively differ from the immune responses of healthy control parents.** PBMCs were stimulated with different classes of antigens (bacteria, fungi, viruses). Cytokines were measured in the cell culture supernatant by ELISA. Comparison of the (A) unstimulated and (B) stimulated general immune response of all BCP-ALL parents and control parents, (C) high-hyperdiploid parents and control parents and (D) ETV6::RUNX1-positive BCP-ALL parents and control parents, further distributed into the (E) anti-bacterial, (F) anti-viral and (G) anti-fungal cytokine responses. Cytokine concentrations were normalized and logtransformed. (A-I) Statistical analysis was performed using Mann-Whitney-Wilcoxon test two-sided with Benjamini-Hochberg correction for multiple testing. A q-value of < 0.05 after correction was considered statistically significant.


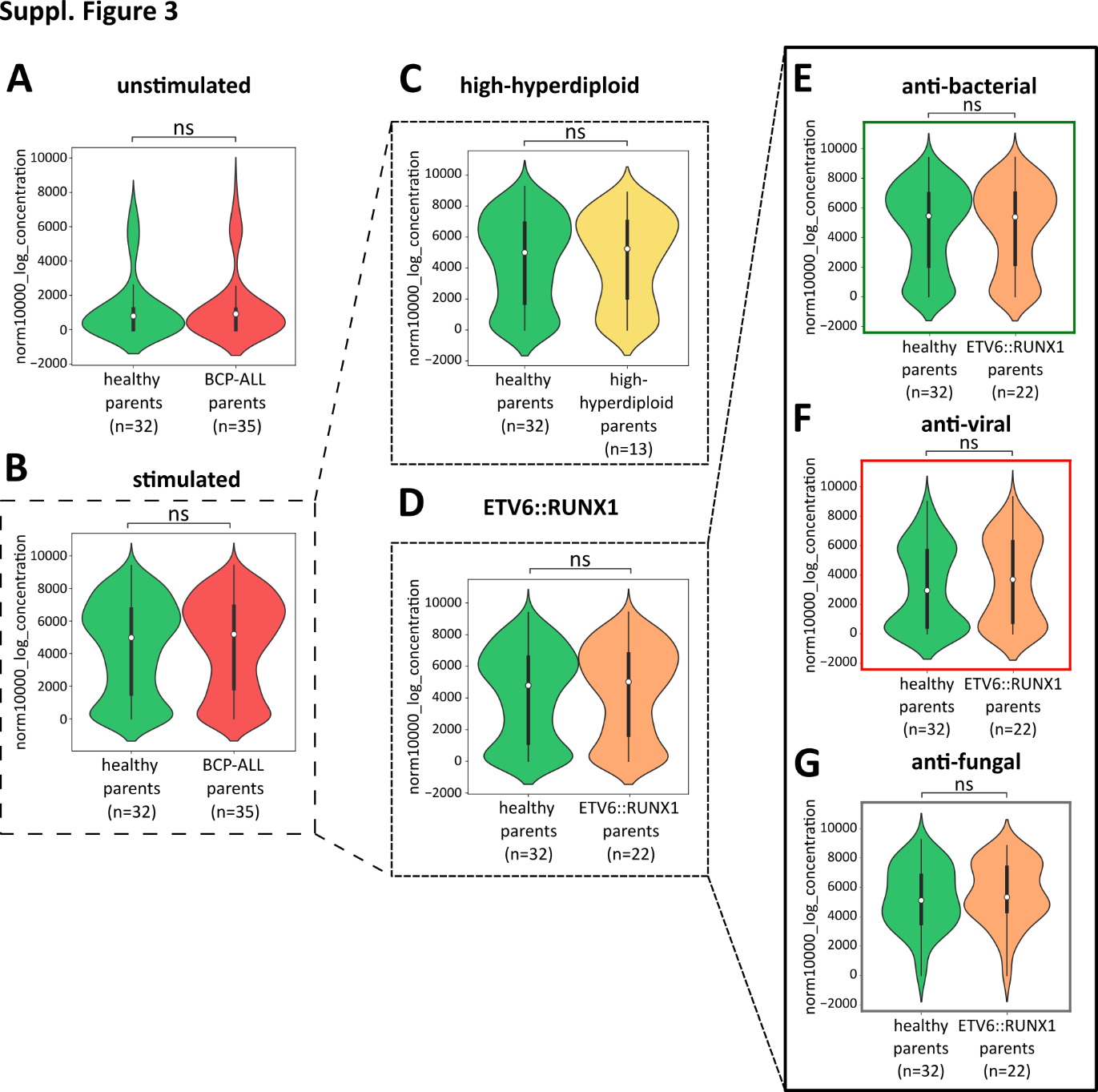


**SDC, Figure. 4: *Ex vivo* stimulation of bone marrow cells from Sca1-ETV6::RUNX1 mice and wild-type mice reveals no differences between the genotypes in Pre-BI and Pre-BII compartments.** (A, B) Apoptosis (n=6), (C, D) proliferation (n=6) and (E, F) IL-7 receptor expression (n=3) were measured in (A, C, E) Pre-BI and (B, D, F) Pre-BII B cell compartments after stimulation with different antigens for 48 h and analyzed using flow cytometry. Statistical analysis was performed using a One-way ANOVA with a Dunnett post hoc test, with a p-value of < 0.05 being considered statistically significant (* = p < 0.05). Mean with SEM is displayed. WT = wild-type.


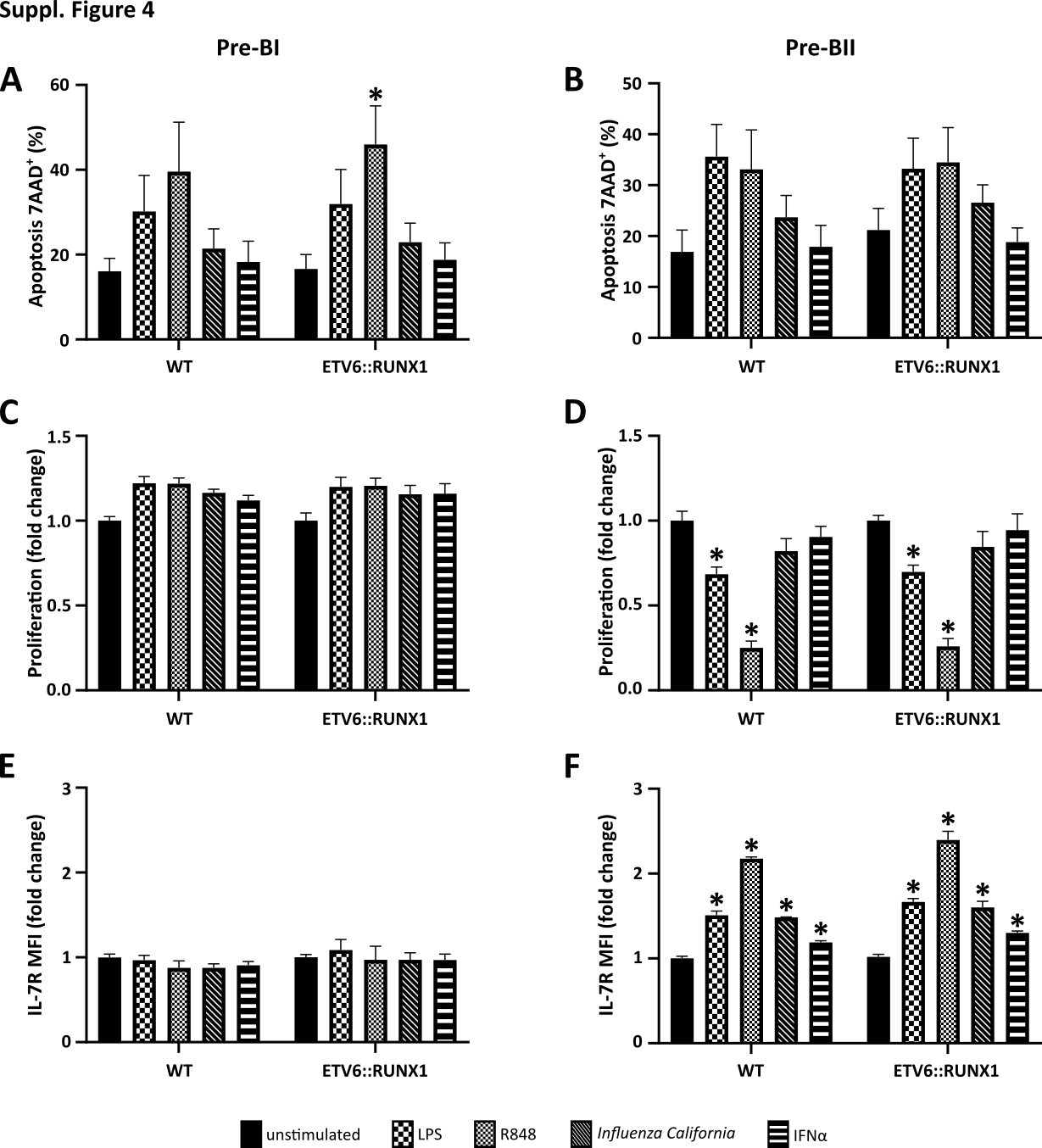


**SDC, Figure. 5: No differences in cytokine profile of bone marrow cells from Sca1-ETV6::RUNX1 mice and wild-type mice could be observed.** *Ex vivo* bone marrow cells were stimulated with different antigens for 24 h and 48 h. Cell culture supernatant was analyzed for (A) murine TNFα (n=6), (B) IFNγ (n=3), (C) IL-6 (n=6) and (D) IL-10 (n=5) levels. Statistical analysis was performed using an unpaired t-test, with a p-value of < 0.05 being considered statistically significant. Mean with SEM is displayed. WT = wild-type, *Infl. California* = *Influenza California.*


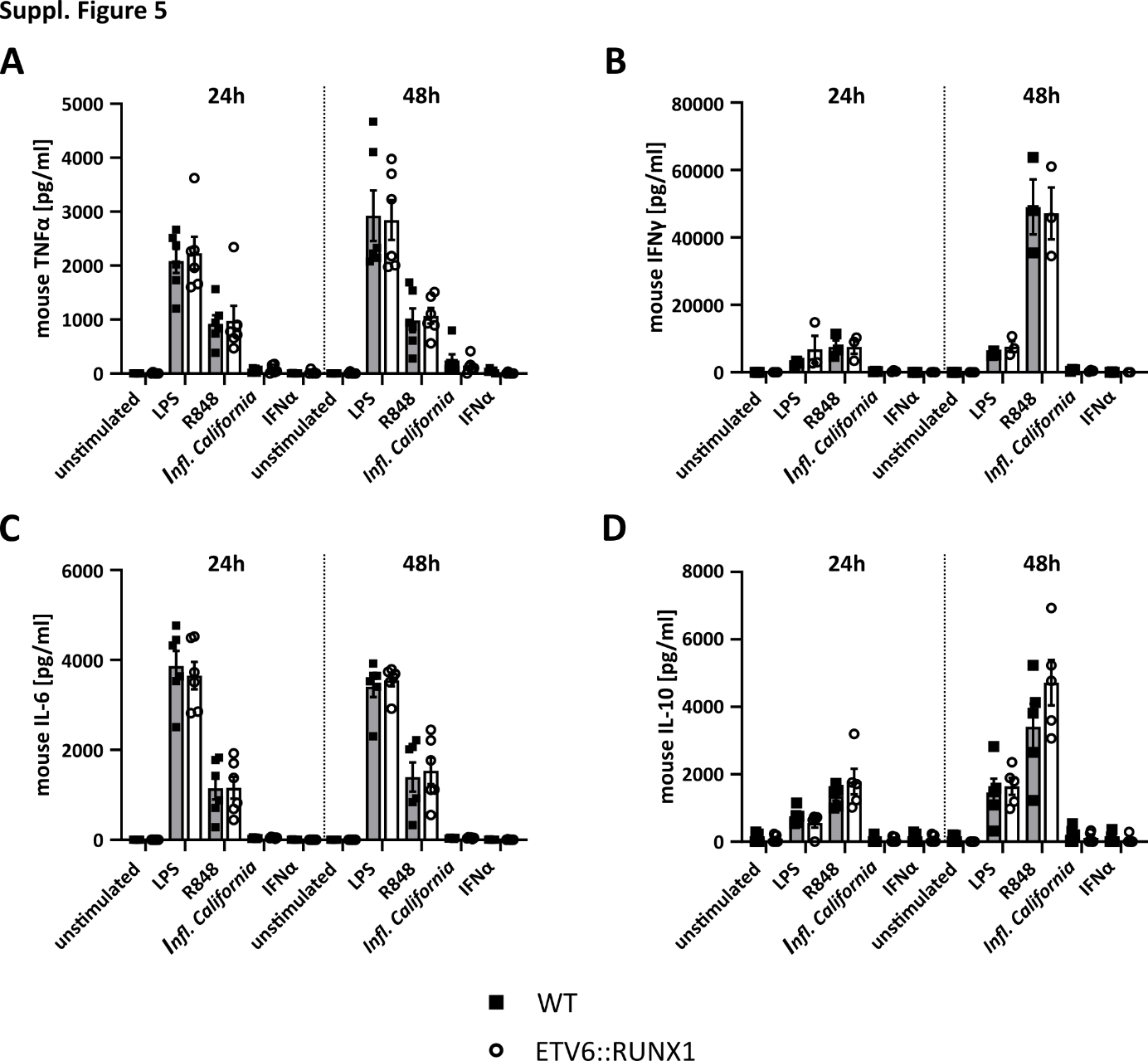


**SDC Figure. 6: Functional validation of NALM-6 cells expressing ETV6::RUNX1.** (A) Western Blot detecting ETV6::RUNX1, RUNX1, RUNX2, RUNX3 and β-Actin as a control gene in the NALM-6 cells stably expressing ETV6::RUNX1 or an empty vector control (B-D) Immunophenotyping of transfected NALM-6 cells after stimulation with PolyI:C and LPS (50 ng/ml and 100 ng/ml) (n=3). (E-F) Cell death (after 24h and 72h) and proliferation (after 72h) of transfected NALM-6 cells after stimulation with IFNα (100 ng/ml and 400 ng/ml), *Influenza California*, R848 and *S. aureus* (n=6 (technical replicates)). Statistical analysis was performed using one sample t tests for the comparison of unstimulated control vs stimulus (in (E) unpaired t tests) and unpaired t tests for the comparison between empty cells and ETV6::RUNX1 expressing cells with a p-value of < 0.05 being considered statistically significant (**** = p < 0.0001, *** = p < 0.001, ** = p < 0.01, * = p < 0.05). Mean with SEM is displayed. *Infl. California* = *Influenza California.*


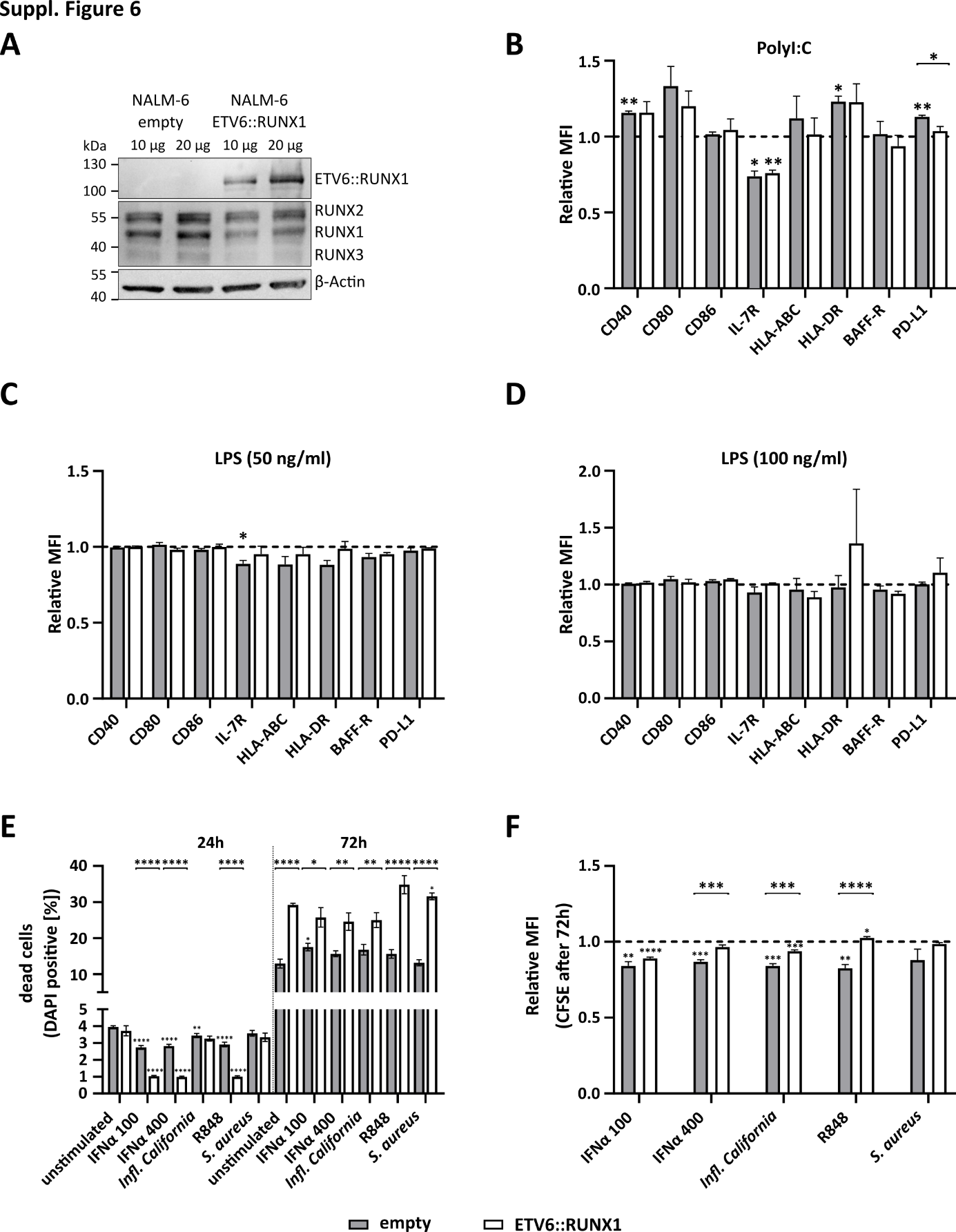


**Additional References:**

Poynton FJ, Thursfield H, Paterson D. The severe blood diseases of childhood: a series of observations from the Hospital for Sick Children, Great Ormond Street. *Br J Child Dis*. 1922;19:128-144.

Martín-Lorenzo A, Hauer J, Vicente-Dueñas C, et al. Infection Exposure Is a Causal Factor in B-cell Precursor Acute Lymphoblastic Leukemia as a Result of Pax5-Inherited Susceptibility. *Cancer Discovery*. 2015;5(12):1328-1343. doi:10.1158/2159-8290.Cd-15-0892

Fischer U, Yang JJ, Ikawa T, et al. Cell Fate Decisions: The Role of Transcription Factors in Early B-cell Development and Leukemia. *Blood Cancer Discov*. Nov 2020;1(3):224-233. doi:10.1158/2643-3230.BCD-20-0011

Hein D, Borkhardt A, Fischer U. Insights into the prenatal origin of childhood acute lymphoblastic leukemia. *Cancer Metastasis Rev*. Mar 2020;39(1):161-171. doi:10.1007/s10555-019-09841-1

Fulbright JM, Raman S, McClellan WS, August KJ. Late effects of childhood leukemia therapy. *Curr Hematol Malig Rep*. Sep 2011;6(3):195-205. doi:10.1007/s11899-011-0094-x

Koskenvuo M, Ekman I, Saha E, et al. Immunological Reconstitution in Children After Completing Conventional Chemotherapy of Acute Lymphoblastic Leukemia is Marked by Impaired B-cell Compartment. *Pediatr Blood Cancer*. Sep 2016;63(9):1653-6. doi:10.1002/pbc.26047

Becker KL, Aimanianda V, Wang X, et al. Aspergillus Cell Wall Chitin Induces Anti- and Proinflammatory Cytokines in Human PBMCs via the Fc-gamma Receptor/Syk/PI3K Pathway. *mBio*. May 31 2016;7(3)doi:10.1128/mBio.01823-15

Fueller E, Schaefer D, Fischer U, et al. Genomic inverse PCR for exploration of ligated breakpoints (GIPFEL), a new method to detect translocations in leukemia. *PLoS One*. 2014;9(8):e104419. doi:10.1371/journal.pone.0104419

Ford AM, Palmi C, Bueno C, et al. The TEL-AML1 leukemia fusion gene dysregulates the TGF-beta pathway in early B lineage progenitor cells. *J Clin Invest*. Apr 2009;119(4):826-36. doi:10.1172/JCI36428

Chang JS, Zhou M, Buffler PA, Chokkalingam AP, Metayer C, Wiemels JL. Profound deficit of IL10 at birth in children who develop childhood acute lymphoblastic leukemia. *Cancer Epidemiol Biomarkers Prev*. Aug 2011;20(8):1736-40. doi:10.1158/1055-9965.EPI-11-0162

Boiers C, Richardson SE, Laycock E, et al. A Human IPS Model Implicates Embryonic B-Myeloid Fate Restriction as Developmental Susceptibility to B Acute Lymphoblastic Leukemia-Associated ETV6-RUNX1. *Dev Cell*. Feb 5 2018;44(3):362-377 e7. doi:10.1016/j.devcel.2017.12.005

Casado-Garcia A, Isidro-Hernandez M, Oak N, et al. Transient Inhibition of the JAK/STAT Pathway Prevents B-ALL Development in Genetically Predisposed Mice. *Cancer Res*. Mar 15 2022;82(6):1098-1109. doi:10.1158/0008-5472.CAN-21-3386

Gallant RE, Arroyo K, Bracci PM, et al. Clinical characteristics of cytomegalovirus-positive pediatric acute lymphoblastic leukemia at diagnosis. *Am J Hematol*. Jun 1 2022;97(6):E198-E201. doi:10.1002/ajh.26528

Haas OA, Borkhardt A. Hyperdiploidy: the longest known, most prevalent, and most enigmatic form of acute lymphoblastic leukemia in children. *Leukemia*. Dec 2022;36(12):2769-2783. doi:10.1038/s41375-022-01720-z

Hein D, Korschgen L, Borkhardt A, Kogler G, Fischer U. Seven Percent of Cord Blood Transplants Carry ETV6-RUNX1 Translocations. *Stem Cells Translational Medicine*. 2020;9(s1):S4-S4. doi:10.1002/sctm.12809

Rodriguez-Hernandez G, Hauer J, Martin-Lorenzo A, et al. Infection Exposure Promotes ETV6-RUNX1 Precursor B-cell Leukemia via Impaired H3K4 Demethylases. *Cancer Res*. Aug 15 2017;77(16):4365-4377. doi:10.1158/0008-5472.CAN-17-0701

Beneforti L, Dander E, Bresolin S, et al. Pro-inflammatory cytokines favor the emergence of ETV6-RUNX1-positive pre-leukemic cells in a model of mesenchymal niche. *Br J Haematol*. Jul 2020;190(2):262-273. doi:10.1111/bjh.16523

Fidanza M, Seif AE, DeMicco A, et al. Inhibition of precursor B-cell malignancy progression by toll-like receptor ligand-induced immune responses. *Leukemia*. Oct 2016;30(10):2116-2119. doi:10.1038/leu.2016.152
